# Pharmacokinetics and Tissue Distribution Study of Pinosylvin in Rats by Ultra-High-Performance Liquid Chromatography Coupled with Linear Trap Quadrupole Orbitrap Mass Spectrometry

**DOI:** 10.1155/2018/4181084

**Published:** 2018-11-21

**Authors:** Yuhang Fu, Xiaoya Sun, Lili Wang, Suiqing Chen

**Affiliations:** ^1^School of Pharmacy, Henan University of Chinese Medicine, No. 156, East Jinshui Road, Zhengzhou, 450046, China; ^2^Collaborative Innovation Center for Respiratory Disease Diagnosis and Treatment & Chinese Medicine Development of Henan Province, Henan University of Chinese Medicine, No. 156, East Jinshui Road, Zhengzhou, 450046, China

## Abstract

Pinosylvin is a potential anti-inflammatory and antioxidant compound and the major effective medicinal ingredient in the root of* Lindera reflexa* Hemsl. However, few investigations have been conducted regarding the pharmacokinetics, excretion, characteristics of tissue distribution, and major metabolites of pinosylvin in rats after oral administration. To better understand the behavior and mechanisms of action underlying the activity of pinosylvin* in vivo*, we established a simple, sensitive, and reliable ultra-high-performance liquid chromatography tandem mass spectrometry (UPLC-MS/MS) method for quantifying pinosylvin in rat plasma, urine, feces, and various tissues (including heart, liver, spleen, lung, kidneys, large intestine, small intestine, and stomach). Noncompartmental pharmacokinetic parameters indicated that pinosylvin is rapidly distributed and taken up by tissues. The time to peak (maximum) concentration (T_max_) was 0.137 h, and the apparent elimination half-life (t_1/2_) was 1.347±0.01 h. The results of the tissue distribution study suggest that pinosylvin is widely distributed to various tissues; the highest concentration was observed after 10 min in the stomach, followed by the heart, lung, spleen, and kidneys. Results of the excretion study suggest that a small amount of pinosylvin is excreted from the urine and feces in the parent form; the 73 h accumulative excretion ratios of urine and feces were 0.82% and 0.11%, respectively. It is likely that pinosylvin is mostly metabolized* in vivo*. Nine metabolites were found, and the main metabolic pathways of pinosylvin in rats included glucuronidation, hydroxylation, and methylation. Four metabolites had higher concentrations in the stomach, suggesting that the stomach is a potential target organ of pinosylvin. In conclusion, the present study may provide a material basis for studying the pharmacological action of pinosylvin and provides meaningful information for the clinical treatment of chronic gastritis and gastric ulcers using Radix Linderae Reflexae.

## 1. Introduction

Pinosylvin (3,5-dihydroxy-trans-stilbene) is a naturally occurring stilbenoid mainly found in the leaves or wood of various* Pinus* species [1, 2] and Lauraceae plants [3, 4]; it is structurally similar to the polyphenol resveratrol. Studies have shown that pinosylvin improves resistance to decay in heartwood. Further, pinosylvin has been shown to have various biological activities, including antifungal and antibacterial [5], cancer chemopreventive/anti-inflammatory [6], cell proliferative, antioxidant [7, 8], vasculo-protective [9], and antiproliferative effects in various cancer cells [10, 11]. Pinosylvin promotes cell proliferation in bovine aortic endothelial cells [7, 12] but inhibits proliferation in human lymphoblastoid cell lines [13].

Radix Linderae Reflexae originates from the root of* Lindera reflexa *Hemsl, which is a new herbal drug listed in the* Dictionary of Chinese Medicine* and has recently been prescribed for the treatment of gastritis and peptic ulcers [14, 15]. Screening experiments for the effective components of Radix Linderae Reflexae showed that the n-butyl alcohol groups significantly improve gastric ulcers. Pinosylvin is included in the n-butyl alcohol groups of Radix Linderae Reflexae [3, 16].

It is well known that pharmacokinetics and characteristics of tissue distribution are vital to understanding* in vivo* behavior and mechanisms of action. To date, a novel and simple high-performance liquid chromatographic method was used for simultaneous determination of pinosylvin in rat serum, and it has been confirmed that plasma levels of pinosylvin decline rapidly after intravenous administration, attributable to a short half-life [17, 18]. However, there are few reports on the pharmacokinetics, excretion, characteristics of tissue distribution, and identification of major metabolites of pinosylvin in rats after oral administration as a single compound.

The goal of this study was to evaluate the metabolic processes associated with pinosylvin in rats and determine target organs by exploring the pharmacokinetics, excretion, characteristics of tissue distribution, and major metabolites after oral administration. This study provides helpful information regarding the clinical study of pinosylvin, as well as traditional Chinese medicines containing pinosylvin.

## 2. Materials and Methods

### 2.1. Chemicals and Reagents

Pinosylvin was isolated in our laboratory from the root of* Lindera reflexa* Hemsl and identified using nuclear magnetic resonance (NMR), mass spectrometry (MS), ultraviolet (UV), and infrared (IR) analyses. Isoliquiritigenin (high-performance liquid chromatography [HPLC] ≥ 98%) was used as the internal standard and purchased from Shanghai Yuanye Bio-Technology Co. Ltd. Heparin sodium was purchased from Beijing Dingguo Changsheng Bio-Technology Co. Ltd. Methanol, acetonitrile, and formic acid were HPLC-grade reagents from Fisher Scientific (Fairlawn, NJ, USA). Deionized water was prepared by passing distilled water through a Milli-Q water purification system (Millipore, Milford, MA, USA).

### 2.2. Instrumentation and Ultra-High-Performance Liquid Chromatography Tandem Mass Spectrometry (UPLC-MS/MS) Conditions

The UPLC-MS/MS system consisted of a Dionex Ultimate 3000 UHPLC system (Thermo Scientific, Germering, Bavaria, Germany) equipped with a binary pump, a thermostatted autosampler, a thermostatically controlled column compartment, a diode array detector (DAD), and a Thermo Fisher LTQ-Orbitrap XL Hybrid Mass Spectrometer with an electrospray ionization (ESI) source. The system control and data analysis were performed using Xcalibur 3.0 software (Thermo Fisher Scientific).

Chromatographic separation was carried out on a reverse-phase Hypersil GOLD C18 column (2.1×50 mm, 1.9 *μ*m) with a UPLC filter (2.1 mm × 0.2 *μ*m) guard column (Thermo Fisher Scientific). The UPLC was operated with a gradient mobile phase system comprising water containing 0.1% formic acid (phase A) and acetonitrile (phase B) at a flow rate of 0.3 ml/min and maintained at 30°C [19]. The pump was programmed as follows: 0.0–1 min, 30% B; 1.0–12.0 min, 30.0–50% B; 12.0–15 min, 50.0–100% B; 15.0–15.1 min, 100.0–30.0% B; 15.1–18.0 min, 30.0%. A 5-*μ*l sample was injected into the system with the autosampler conditioned at 7°C.

**Figure 1:**
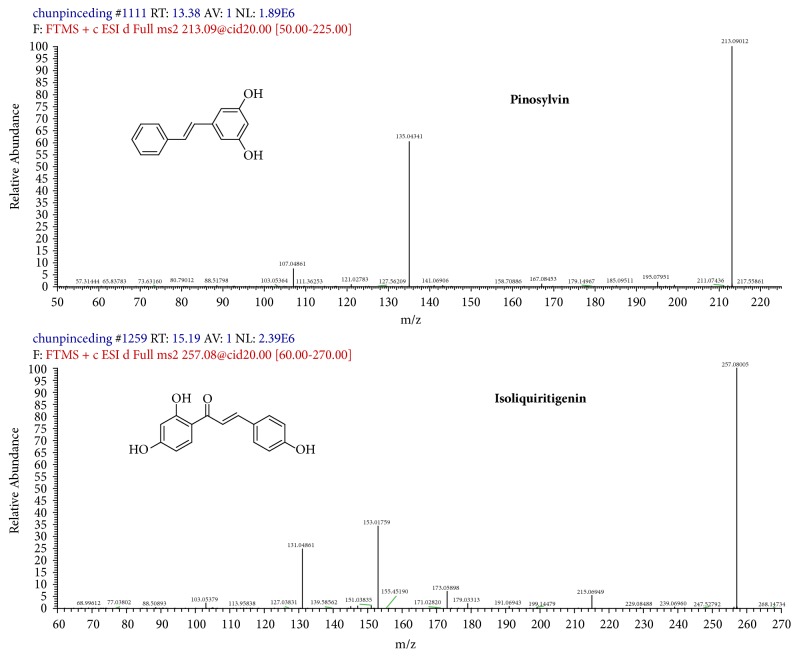
Chemical structures and mass spectra of pinosylvin and isoliquiritigenin (IS).

The mass spectrometer was operated in positive ion mode. Selected ion monitoring (SIM) was used, and the fragmentation transitions were m/z 213.09 for pinosylvin and m/z 257.08 for isoliquiritigenin (Figure 1). The ESI source parameters were set as follows: ion spray voltage, 4200 V; capillary temperature, 350°C; capillary voltage, 23 V; and tube lens voltage, 90 V. The flow rates of sheath (N_2_) and auxiliary gas (He) were 40 and 10 arbitrary units, respectively.

### 2.3. Collection and Treatment of the Plasma, Urine, Feces, and Tissues

Sprague-Dawley (SD) male rats weighing 180–220 g were provided by Henan Experimental Animal Center (Zhengzhou, China). All rats were kept in an environmentally controlled breeding room maintained at a temperature of 22 ± 2°C with relative humidity of 50% and were fed standard laboratory food and water for one week prior to the experiments. All rats were fasted overnight with access to water only before experiments.

Blood samples were obtained from the rat orbital vein and placed into centrifuge tubes containing heparin sodium (20 *μ*l, 1%). The blood samples were immediately centrifuged at 10000 rpm for 10 min (4°C), and the supernatant was gathered as the plasma. Urine and feces were collected in metabolic cages and packed separately with centrifuge tubes as samples [20]. Various tissues (including heart, liver, spleen, lung, kidney, large intestine, small intestine, and stomach) were harvested and rinsed with ice-cold 0.9% NaCl to remove the superficial blood and contents. After being blotted dry with filter paper, certain equal amounts of tissues were accurately weighed and homogenized in 0.9% NaCl to prepare the homogenates (1:2, m/v) [21].

### 2.4. Standard and Sample Preparation

#### 2.4.1. Preparation of Stock and Working Solutions

The stock solutions were prepared by dissolving pinosylvin and isoliquiritigenin in methanol to reach a final concentration of 5.40 mg/mL and 0.55 mg/mL, respectively. The stock working solution of pinosylvin was serially diluted with methanol to a linear concentration of 0.0027–1728.0000 *μ*g/ml. An isoliquiritigenin solution (IS, 100 ng/ml) was prepared in methanol. All solutions were stored at 4°C in the dark.

#### 2.4.2. Preparation of Calibration Standards and Quality Control (QC) Samples

Calibration curves were prepared by spiking the standard working stock solutions with 10 *μ*l of the different concentrations, 20 *μ*l IS (110 ng/ml), and 90 *μ*l blank rat plasma, urine, feces, or tissue homogenate sample in a 1.5-ml centrifuge tube on the analysis day. The calibration standards were prepared at concentrations of 0.0027, 0.0054, 0.1080, 0.1350, 0.2700, and 0.5400 *μ*g/ml for the plasma; 0.0027, 0.5400, 1.3500, 2.7000, 5.4000, 13.5000, 27.0000, and 54.0000 *μ*g/ml for urine; 0.1350, 0.2700, 1.3500, 2.7000, 5.4000, and 27.0000 *μ*g/ml for the feces, spleen, and kidney; 0.2700, 0.5400, 1.3500, 2.7000, 5.4000, 13.5000, 27.0000, and 54.0000 *μ*g/ml for the heart; 1.3500, 2.7000, 13.5000, 27.0000, 54.0000, and 108.0000 *μ*g/ml for the liver and large intestine; 0.5400, 1.3500, 2.7000, 5.4000, 13.5000, and 27.0000 *μ*g/ml for the lung; 2.7000, 5.4000, 13.5000, 27.0000, 54.0000, 108.0000, 216.0000, and 432.0000 *μ*g/ml for the small intestine; 1.3500, 2.7000, 5.4000, 13.5000, 27.0000, 54.0000, 108.0000, 216.0000, 432.0000, 864.0000, and 1728.0000 *μ*g/ml for the stomach. Quality control (QC) samples were prepared in the same way with blank plasma, urine, feces, or tissue homogenates at concentrations of 0.0054, 0.1350, and 0.5400 *μ*g/ml for the plasma; 0.2700, 2.7000, and 5.4000 *μ*g/ml for the feces; 1.3500, 5.4000, and 54.0000 *μ*g/ml for the urine; 0.5400, 5.4000, and 27.0000 *μ*g/ml for the heart; 2.7000, 13.5000, and 54.0000 *μ*g/ml for the liver and large intestine; 0.2700, 5.4000, and 13.5000 *μ*g/ml for the spleen; 0.5400, 2.7000, and 13.5000 *μ*g/ml for the lung; 0.5400, 5.4000, and 13.5000 *μ*g/ml for the kidney; 5.4000, 108.0000, and 216.0000 *μ*g/ml for the small intestine; 13.5000, 216.0000, and 864.0000 *μ*g/ml for the stomach. Moreover, the concentration of IS in all samples was 110.0000 ng/ml.

#### 2.4.3. Sample Treatment

A simple liquid-liquid extraction (LLE) method was used to extract pinosylvin from QC samples, calibration standards, and all biosamples (including plasma samples, urine samples, fecal samples, and tissue homogenate samples). After biosamples were thawed at room temperature, 100-*μ*l aliquots were transferred to 1.5-ml tubes. The samples were first vortex-mixed with IS (10 *μ*l, 110 ng/ml) and then with a solution of methanol-acetonitrile (0.9 ml, 5:95, v/v) for extraction. After vortexing for 5 min and centrifuging at 10000 rpm, 4°C for 10 min. The supernatant (900 *μ*l) was transferred to a new 1.5-ml centrifuge tube and evaporated to dryness under vacuum. The dried residue was reconstituted with 50 *μ*l methanol, vortex-mixed at 2000 k for 5 min, and centrifuged at 16000 rcf (4°C for 10 min). Finally, the supernatant liquid (5 *μ*l) was injected into the UPLC-ESI-MS/MS system.

### 2.5. Method Validation

The developed method was validated according to the China Food and Drug Administration (CFDA) technical guidelines for the study of nonclinical pharmacokinetics.

#### 2.5.1. Specificity

Specificity of the method was assessed by analyzing blank biological samples from at least six different sources (plasma, urine, feces, and various tissue homogenate samples), blank biological matrix samples spiked with pinosylvin and IS, and actual biosamples after oral administration of pinosylvin and spiking with IS.

#### 2.5.2. Linearity and Sensitivity

The calibration standards of pinosylvin were in the concentration range of 0.0027–0.5400 *μ*g/ml for plasma samples; 0.0027–54.0000 *μ*g/ml for urine samples; 0.1350–27.0000 *μ*g/ml for feces, and 0.1350–1728.0000 *μ*g/ml for tissue samples. Calibration curves were established by plotting the peak area ratios of the analytes to IS (Y-axis) versus the nominal concentration of pinosylvin (X-axis) using weighted least-squares linear regression analysis.

The lowest concentration on the calibration curve was set as the lower limit of quantification (LLOQ), and we determined the drug concentration in the sample for at least 3–5 half-lives as required. The precision and accuracy of LLOQ should not exceed 20%.

#### 2.5.3. Precision and Accuracy

Precision and accuracy were assessed with the QC samples (low, middle, and high concentration) in five replicates prepared and analyzed on three consecutive days. Intraday and interday precision was evaluated with relative standard deviation (RSD%) values. To assess the accuracy, the relative error (RE%) was calculated according to the following formula: RE% = [(assayed value - normal value)/normal value]×100%. An accuracy within ±15% of the RE and a precision ≤ 15% of the RSD (all of them near the lower limit and should be less than 20%) were deemed acceptable.

#### 2.5.4. Extraction Recovery and Matrix Effects

The extraction recoveries in rat sample matrices for pinosylvin and the IS were calculated as the peak area ratios of the rat sample matrix spiked with a standard solution to the blank matrix spiked with an equivalent standard solution. The recovery of pinosylvin was determined at low, medium, and high concentrations, while the recovery of the IS was determined at a single concentration of 110 ng/ml.

The matrix effect of extraction on pinosylvin was evaluated by comparing the peak areas of the methanol-acetonitrile (5:95, v/v) extracted blank samples spiked with pinosylvin at three QC concentrations with those of the pinosylvin standard solution at equivalent concentrations.

#### 2.5.5. Stability

Stability was investigated by analyzing five replicates of the samples at three QC levels under different conditions, including storage for 24 h in the autosampler, three freeze/thaw cycles, storage for 12 h at ambient temperatures (25°C), and storage at -80°C for 30 days. The samples were considered stable if the average percentage concentration deviation was within 15% of the actual value.

### 2.6. Pharmacokinetic Study

For the pharmacokinetic study, blank blood samples were collected from the orbital vein of rats using sterile capillary tubes. After oral administration of pinosylvin (49.44 mg/kg dissolved in 0.1% sodium carboxymethyl cellulose) to SD rats (n=6), approximately 200 *μ*l blood was collected into heparinized tubes at 8 min, 10 min, 20 min, 30 min, 45 min, 60 min, 80 min, 100 min, 2 h, 4 h, 6 h, 12 h, and 24 h. The samples were immediately centrifuged at 10000 rpm for 10 min and 4°C, and the supernatant plasma layer (100 *μ*l) was transferred to a new 1.5-ml centrifuge tube and stored at -80°C until analysis.

### 2.7. Excretion Study

For the excretion study, blank urine samples and fecal samples were collected using metabolic cages. After oral administration of pinosylvin (49.44 mg/kg dissolved in 0.1% sodium carboxymethyl cellulose) to SD rats (n=6), urine and feces were collected at 0–2 h, 2–4 h, 4–8 h, 8–10 h, 10–12 h, 12–24 h, 24–36 h, 36–48 h, 48–60 h, and 60–73 h. Urine volumes were recorded, and 100 *μ*l was transferred to centrifuge tubes for use as the urine samples. Fecal weights were recorded after drying in a dark environment; the resulting powders (0.05 g) were added to centrifuge tubes and then mixed with NaCl (200 *μ*l, 0.9% solution) for use as the fecal samples. All the samples were stored at -80°C until analysis.

### 2.8. Tissue Distribution Study

For the tissue distribution study, 36 rats were randomly assigned to six groups (6 rats/group) and sacrificed at 10 min, 20 min, 1 h, 2 h, 6 h, and 8 h after orally administering pinosylvin (49.44 mg/kg dissolved in 0.1% sodium carboxymethyl cellulose). Subsequently, the heart, liver, spleen, lungs, kidneys, large intestine, small intestine, and stomach were immediately removed, washed in normal saline, and blotted dry with filter paper. An accurately weighed amount of fresh tissue sample (0.25 g) was individually homogenized with normal saline (0.5 ml) and transferred (100 *μ*l) as a tissue homogenate to 1.5-ml centrifuge tubes for use as the tissue samples. All tissue samples were stored at -80°C until analysis.

### 2.9. Analysis of Metabolites

We used MetWorks™ 1.3 SP4 software (Henan University of Chinese Medicine, Zhengzhou, China) to analyze the metabolite and biosample data collected via Xcalibur 3.0 software (Thermo Fisher Scientific); the aim was to further describe the metabolite profiles of pinosylvin in rat plasma and tissue.

### 2.10. Data Analysis

DAS 3.2.8 software (Henan University of Chinese Medicine, Zhengzhou, China) was used to calculate the pharmacokinetic parameters using a noncompartmental model, including half-life (t_1/2_), area under the curve (AUC), apparent central volume of mean residual time (MRT), and clearance rate (CL). All other results are expressed as means ± SD. Concentrations of pinosylvin in rat urine and feces were calculated according to their respective calibration curves using the ratio of their peak area to that of the IS using the following equation: excretion ratio = [measured concentration × volume (weight)/dosage]×100%.

## 3. Results and Discussion

### 3.1. Optimization of UPLC-MS/MS Conditions and Extraction Method

The LTQ-Orbitrap conditions were systematically optimized; full scan was used in the positive and negative detection mode after individually injecting approximately 540 ng/ml pinosylvin in methanol and 110 ng/ml IS in methanol. The results show that sensitivity was higher for pinosylvin and IS when analyzed in the positive ion mode. Pinosylvin and isoliquiritigenin predominantly gave a singly charged protonated precursor [M+H]^+^ at m/z 213.09 and 257.08 in Q1 full scan mode, respectively. Therefore, selected ion monitoring (SIM) was used, and the fragmentation transitions were m/z 213.09 for pinosylvin and m/z 257.08 for isoliquiritigenin.

The UPLC conditions, including the mobile phase systems and the type of chromatographic columns. The results show that acetonitrile gave a better peak shape and lower background noise than methanol as the organic phase. Further, pinosylvin and IS had a higher response when the water phase contained 0.1% formic acid. Retention times for both pinosylvin and IS were less than 5 min when using the Hypersil GOLD C18 column with a shorter length (50 mm) when the mobile phase was set at a flow rate of 0.3 ml/min. These parameters improved the speed of sample analysis.

The solid phase extraction column was first considered for use in sample preparation; however, the extraction recovery did not meet the analytical requirements. Therefore, a liquid-liquid extraction (LLE) method was chosen for sample preparation. We found that methanol-acetonitrile (5:95, v/v) was the best choice as an extraction solvent, yielding a higher extraction ratio and lower background interference.

### 3.2. Method Validation

#### 3.2.1. Selectivity

**Figure 2:**
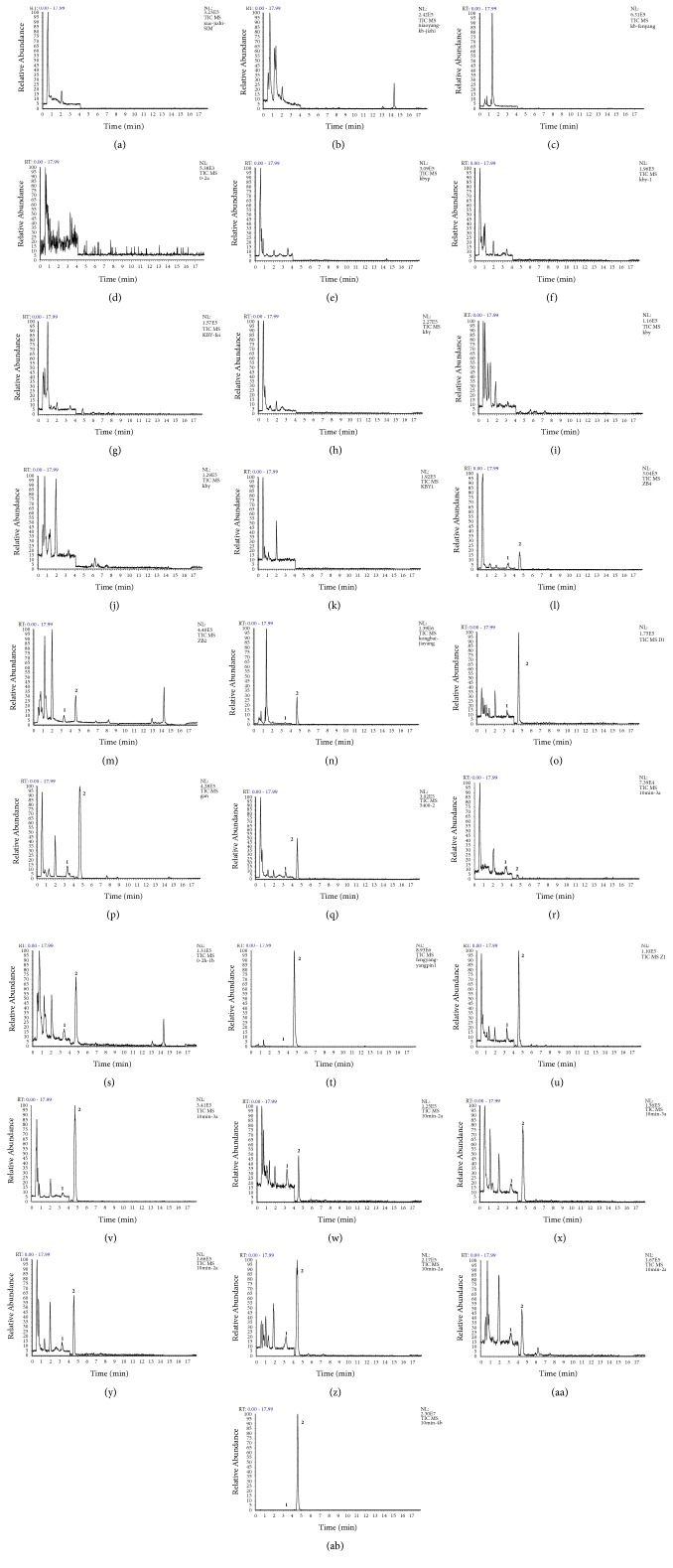
Representative selected ion monitoring (SIM) chromatograms of blank plasma (a), blank urine (b), blank feces (c), blank heart (d), blank liver (e), blank spleen (f), blank lung (g), blank kidney (h), blank large intestine (i), blank small intestine (j), blank stomach (k), plasma spiked with pinosylvin and isoliquiritigenin solution (IS) (l), urine spiked with pinosylvin and IS (m), feces spiked with pinosylvin and IS (n), stomach spiked with pinosylvin and IS (o), liver spiked with pinosylvin and IS (p), kidney spiked with pinosylvin and IS (q), plasma sample (r) obtained 10 min after oral administration of 49.44 mg/kg pinosylvin, urine sample (s) obtained 0–2 h after oral administration of 49.44 mg/kg pinosylvin, feces sample (t) obtained 4–6 h after oral administration of 49.44 mg/kg pinosylvin, and heart sample (u), liver sample (v), spleen sample (w), lung sample (x), kidney sample (y), large intestine sample (z), small intestine sample (aa), stomach sample (ab) obtained 10 min after oral administration of 49.44 mg/kg pinosylvin. Peak 1 reflects IS, and peak 2 reflects pinosylvin.

Due to the high selectivity and specificity of SIM mode by the linear trap Quadrupole Orbitrap Mass Spectrometer, no endogenous interference was observed at retention times of 4.70 min (pinosylvin) or 3.46 min (IS). Typical chromatograms of blank plasma, urine, feces, and stomach homogenates; blank plasma, urine, feces, stomach, and liver spiked with pinosylvin and IS; and all biosamples after oral administration of pinosylvin are presented in Figure 2.

#### 3.2.2. Calibration Curves and LLOQ

Calibration curves used to determine coefficients and linear ranges of pinosylvin in plasma, urine, feces, and each tissue are listed in Table 1. Further, calibration curves for all matrices showed good linearity (r>0.9916) over the concentration ranges. LLOQs were 0.0027 *μ*g/ml (S/N>10) for plasma and urine; 0.2700 *μ*g/ml for heart; 0.1350 *μ*g/ml for feces, spleen, and kidney; 1.3500 *μ*g/ml for liver, stomach, and large intestine; 0.5400 *μ*g/ml for lung; and 2.7000 *μ*g/ml for small intestine.

#### 3.2.3. Precision and Accuracy

The results for intra- and interday precision and accuracy at three QC concentrations are presented in Table 2. The intra- and interday accuracy were within -10.8 % to 11.6%, respectively, while the intra- and interday precision were less than 14.7% and within the acceptable criteria of ±15%, indicating that the precision and accuracy of this assay were within acceptable ranges for analysis.

#### 3.2.4. Extraction Recovery and Matrix Effect

The extraction recovery and matrix effect for pinosylvin are shown in Table 3. All extraction recoveries of pinosylvin (at three concentration levels) and IS (at 110 ng/ml) in biosamples ranged from 83.3% to 108.5% with a RSD% less than 13.3%, demonstrating that the extraction method was acceptable and met the requirements of analysis. The absolute matrix effect values of pinosylvin ranged from 81.3% to 114.8%, with a RSD% less than 13.5%. These results indicate that, for this UPLC-MS/MS determination, there were no significant matrix effects for pinosylvin in the plasma, urine, feces, or tissue samples.

#### 3.2.5. Stability

Stability was tested under various conditions that might occur during sample preparation. The results shown in Table 4 demonstrate that pinosylvin was stable in rat plasma, urine, feces, and tissue homogenates after storage for 24 h in the autosampler, three freeze/thaw cycles, storage for 12 h at ambient temperature, and storage at -80°C for 30 days.

### 3.3. Pharmacokinetic Study

**Figure 3:**
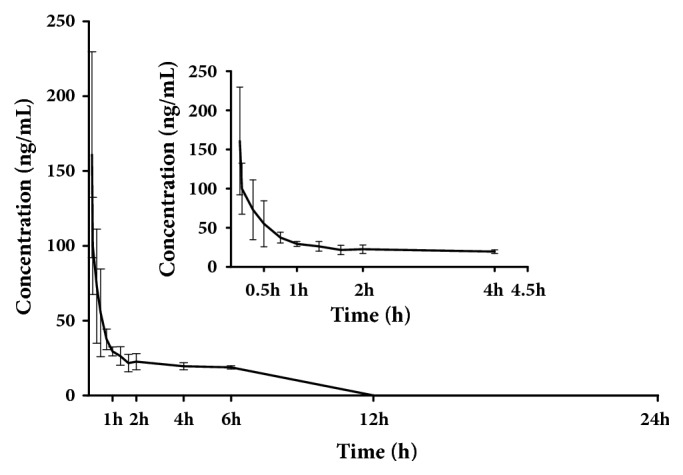
Mean plasma concentration-time curve of pinosylvin after oral administration of 49.44 mg/kg pinosylvin (n=6, mean ± SD).

The validated UPLC-MS/MS method was successfully used to investigate the pharmacokinetics of pinosylvin after oral administration at a dose of 49.44 mg/kg. The mean plasma concentration-time curves are shown in Figure 3. The corresponding pharmacokinetic parameters calculated with noncompartmental analysis are listed as means ± SD and shown in Table 5.

The results show that the time to peak (maximum) concentration (T_max_) was 0.137 h after oral administration in rats, and the peak (maximum) plasma concentration (C_max_) of pinosylvin was 164.231 ± 64.264 ng/ml. The plasma concentration of pinosylvin decreased sharply from 160.929 ng/ml to 22.581 ng/ml after 2 h. The apparent elimination half-life (t_1/2_) was 1.347 ± 0.01 h, indicating pinosylvin was quickly cleared from the rat plasma. The AUC_0-24h_ and AUC_0-*∞*_ were 711.142 ± 46.885 and 711.195 ± 46.885, respectively. The apparent volume of distribution (V_d_) results imply that pinosylvin is taken up by the tissues after oral administration [18, 22, 23].

To our knowledge, there are no reports regarding the pharmacokinetics of pinosylvin administered orally as a single compound. In this study, we investigated the pharmacokinetics of pinosylvin in rats to determine its pharmacokinetic behavior* in vivo*. These results will provide helpful information for the clinical treatment of chronic gastritis and gastric ulcers using Radix Linderae Reflexae.

### 3.4. Tissue Distribution Study

**Figure 4:**
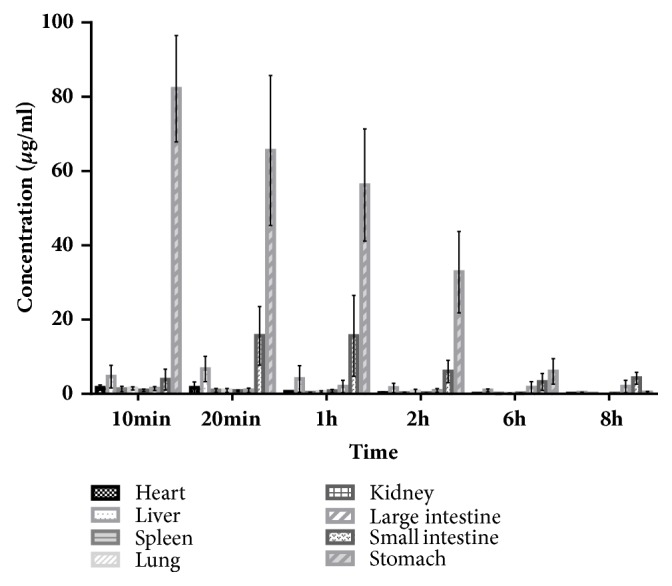
Mean concentration of pinosylvin in various tissues (including heart, liver, spleen, lungs, kidneys, large intestine, small intestine, and stomach) 10 min, 20 min, 1 h, 2 h, 6 h, and 8 h after oral administration of 49.44 mg/kg pinosylvin (n=6, mean ± SD).

Pinosylvin distributions to the heart, liver, spleen, lungs, kidneys, large intestine, small intestine, and stomach are listed in Figure 4. Pinosylvin was widely distributed in various tissues, and the highest concentration was observed at 10 min in the stomach, followed by the heart, lungs, spleen, and kidneys. While the highest concentration was observed in the liver at 20 min, high concentrations remained in the small intestine from 20 min to 6 h after oral administration. These results demonstrate enterohepatic circulation of pinosylvin in rats. Pinosylvin was more concentrated in the tissue than the plasma, suggesting that pinosylvin is rapidly divided into various target organs after oral administration.

To date, there have been few pharmacokinetic or tissue distribution studies of pinosylvin after oral administration. In contrast, there have been many reports regarding resveratrol, which has a similar structure to that of pinosylvin; these reports mainly focus on cellular aspects. As a potential anti-inflammatory compound [24, 25], pinosylvin is rapidly distributed to the stomach where it persists over time, suggesting that it may be an effective component of Radix Linderae Reflexae for the treatment of chronic gastritis and gastric ulcers.

### 3.5. Excretion Study

**Figure 5:**
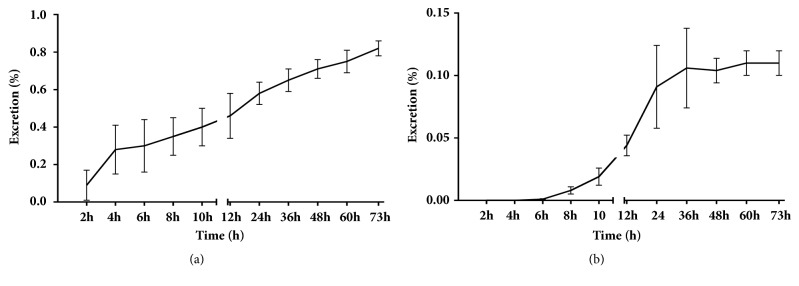
Accumulative excretion ratio of pinosylvin in urine (a) and feces (b) after oral administration of 49.44 mg/kg pinosylvin (n=6, mean ± SD).

Results from the excretion study of urine and feces are shown in Figure 5. The 73-h accumulative excretion ratios of urine and feces were 0.82% and 0.11%, respectively. The excretion peak of pinosylvin in urine samples was noted 2–4 h after oral administration. After 24 h, a small amount of pinosylvin was detected in the urine. Similar to the urine excretion data, pinosylvin was rapidly excreted from the feces in the parent form from 6 to 24 h after oral administration. It is likely that pinosylvin is mostly metabolized* in vivo* and plays a role in different organs.

### 3.6. Metabolite Identification Study

**Figure 6:**
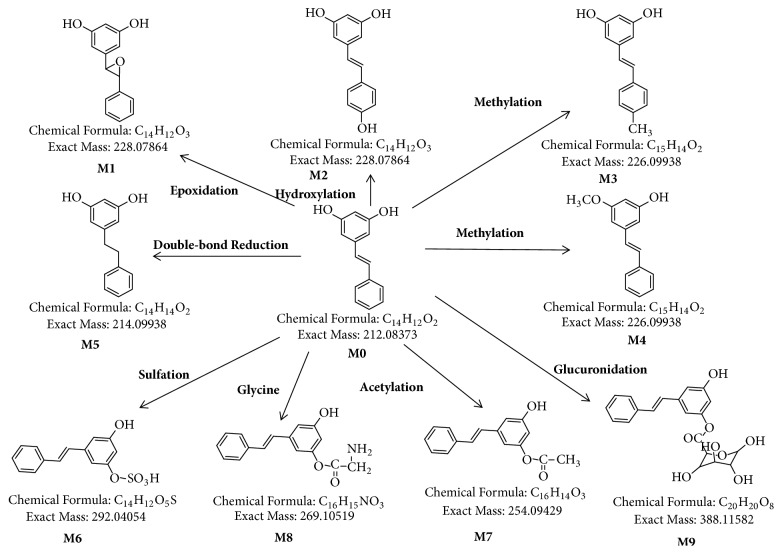
Proposed metabolic pathways in rats after oral administration of 49.44 mg/kg pinosylvin.

In this study, 9 possible metabolites were found in rats according to the full-scanning mass spectrograms of all biosamples and the characteristics of phase I and phase II, which are shown in Table 6. The metabolic processes in rats are complex. Therefore, it is difficult to determine the exact metabolic pathways of parent compounds into the respective metabolites. As such, metabolic pathways can only be speculated [26, 27]. The proposed metabolic pathways of pinosylvin in rats are shown in Figure 6.

The liver is the main metabolic organ of pinosylvin in rats [18, 28, 29], and all metabolites were detected in the liver except M8. However, M8 was more concentrated in the urine and fecal samples than the other metabolites examined. M9 was the main phase II metabolite and was widely found in all biosamples except urine. The stomach is a potential target organ of pinosylvin, and all measured metabolites were detected in the stomach after oral administration. The main metabolites detected in the heart were M7 and M9, whereas M1, M2, M5, and M9 were detected in samples from the large and small intestines. As a phase I metabolite of pinosylvin, M6 was more concentrated in the kidney than in other organs. These results could provide references for the further development of pinosylvin.

## 4. Conclusions

In the present study, a simple, sensitive, and reliable UPLC-MS/MS method for the quantification of pinosylvin in rat plasma, urine, feces, and various tissues (including heart, liver, spleen, lungs, kidneys, large intestine, small intestine, and stomach) was established. This method was validated with good specificity, linearity, precision, accuracy, and extraction; therefore, it was successfully used to evaluate the pharmacokinetics, excretion, and tissue distribution of pinosylvin in rats. As a potential anti-inflammatory compound, pinosylvin was cleared quickly from rat plasma within 2 h after a single oral administration of 49.44 mg/kg. Within 6 h after oral administration, the concentration of pinosylvin in the stomach was at the highest level. A small amount of pinosylvin was excreted from the urine and feces, indicating that most of the parent drug (pinosylvin) was metabolized* in vivo*. Nine metabolites were found in the samples, and the main metabolic pathways for pinosylvin in rats included glucuronidation, hydroxylation, and methylation. Four metabolites had higher concentrations in the stomach than other organs, suggesting that the stomach is a potential target organ of pinosylvin.

Pinosylvin is a main chemical constituent of the effective component of Radix Linderae Reflexae. This study evaluated the metabolic processes of pinosylvin in rats. The results provide valuable information regarding the clinical treatment of chronic gastritis and gastric ulcer with Radix Linderae Reflexae.

## Figures and Tables

**Table 1:** Calibration curve, correlation coefficients, and linear ranges of pinosylvin in different biosamples.

Samples	Calibration Curve	Correlation Coefficient (r)	Linear range (*μ*g/ml)
Plasma	Y=0.9749X+0.1711	0.9997	0.0027-0.5400
Urine	Y=1.4788X+1.1261	0.9936	0.0027-54.0000
Feces	Y=0.6249X-0.3637	0.9995	0.1350-27.0000
Heart	Y=2.8938X+0.3503	0.9964	0.2700-54.0000
Liver	Y=4.3646X-0.3492	0.9974	1.3500-108.0000
Spleen	Y=2.2020X+0.2676	0.998	0.1350-27.0000
Lung	Y=1.6875X+0.3068	0.9995	0.5400-27.0000
Kidney	Y=1.5383X+0.1088	0.9987	0.1350-27.0000
Large intestine	Y=0.9523X+1.0799	0.9916	1.3500-108.0000
Small intestine	Y=2.3900X+15.9520	0.9932	2.7000-432.0000
Stomach	Y=3.3719X-7.1728	0.9968	1.3500-1728.0000

**Table 2:** Intra- and interassay precision and accuracy for determining pinosylvin in rat plasma, urine, feces, and various tissue homogenates (n=3 days, 5 replicates per day).

Bio-sample	Nominal concentration (ng/mL)	Inter-day (n=5)		Intra-day (n=15)	
		Precision RSD (%)	Accuracy R.E (%)	Precision R.S.D (%)	Accuracy R.E (%)
Plasma	5.4	2.7	7.5	3.7	9.4
	135	10	-6.1	10.1	-7.4
	540	4.8	0.2	9.4	6.5
Urine	1350	0.7	9.5	1.7	9.9
	5400	8.6	-7.4	8	-7.8
	54000	6.4	-6.3	4.5	-2.9
Feces	270	8.6	-5.4	9.8	2.4
	2700	8.3	-3.6	9.6	-1.1
	5400	9.3	-3.5	9.5	-5.9
Heart	540	9.1	-4.7	9.8	6.4
	5400	4.4	2.1	7.3	4.1
	27000	3	10	3	9.6
Liver	2700	6.6	-4.8	9	-5.3
	13500	0.8	0.09	9.5	7
	54000	4.6	8.4	5.1	9.7
Spleen	270	1.2	9.9	1.7	11.6
	5400	7.9	0.2	8.9	4.8
	13500	9.2	-8.7	9.8	-7.3
Lung	540	6.1	9.8	6.4	10.7
	2700	8.6	-2.5	9.6	3
	13500	6.5	9	8.6	11
Kidney	540	6	-7.9	9.5	-8.5
	5400	5.2	-3.1	6.1	-3.9
	13500	7.6	-10.8	9.1	-8.7
Large intestine	2700	12.4	-8.6	11.9	-1.4
	13500	6.5	-9.4	10	-7.5
	54000	4.1	3.4	7.1	2
Small intestine	5400	2.2	9	10.7	8.9
	108000	8.4	1.1	9.7	2.3
	216000	8.6	1.7	7.2	7.3
Stomach	13500	14.3	7.5	13.5	4.6
	216000	13.1	-0.2	14.2	2.4
	864000	12.1	-8.5	14.7	-4.8

**Table 3:** Extraction recovery and matrix effect of pinosylvin in rat plasma, urine, feces, and various tissue homogenates (n=5).

Bio-sample	Nominal concentrations (ng/mL)	Extraction recovery (%)	RSD (%)	Matrix effect (%)	RSD (%)
Plasma	5.4	98.8±7.1	8	99.1±8.5	8.6
	135	95.2±2.5	3	103.3±5.4	5.8
	540	99.5±3.7	4.2	114.8±3.3	3.2
Urine	1350	91.6±8.0	9.8	110.3±10.6	10.8
	5400	108.5±2.5	2.6	81.3±5.3	7.3
	54000	98.3±6.8	7.7	107.2±9.2	9.6
Feces	270	83.3±7.3	9.7	107.8±7.7	8
	2700	99±6.1	7	98.2±6.7	7.6
	5400	96.4±4.4	5.1	97.8±5.9	6.8
Heart	540	89.8±5.1	6.3	103.7±6.8	7.3
	5400	99.3±5.3	6	94.1±7.1	8.4
	27000	99.8±2.6	2.9	96.9±6.5	7.5
Liver	2700	86.5±4.8	6.3	103.2±4.7	5.1
	13500	83.4±5.5	7.4	88.4±7.9	10
	54000	87.1±3.8	4.9	89.3±5.5	6.9
Spleen	270	91.1±7.3	9	89.7±7.9	9.8
	5400	93.5±7.3	8.7	94.8±7.3	8.7
	13500	93.7±5.2	6.2	93.7±8	9.6
Lung	540	98.4±6.4	7.3	88.5±5.3	6.8
	2700	91.5±6.7	8.2	91.6±7.8	9.4
	13500	86±4	5.2	90.5±6.9	8.5
Kidney	540	86.5±6.1	7	98.4±8	8.2
	5400	99.6±4.8	4.9	101.8±6.2	6.1
	13500	90.5±6.4	7	92.6±9.2	9.9
Large intestine	2700	96.5±8.3	9.6	107.1±9.4	9.8
	13500	98.6±5	5.7	99±6.8	7.7
	54000	98.9±5	5.6	93.0±7.6	9.1
Small intestine	5400	89.4±10.7	13.3	88.7±7.7	9.7
	108000	88.6±6	7.5	89.4±8	10
	216000	92.9±7.5	9.1	83.2±1.3	1.7
Stomach	13500	87.5±10.1	13	90.8±7.1	8.8
	216000	89.9±7.7	9.6	94±11.4	13.5
	864000	86.3±9.2	12	92.6±9	10.8

**Table 4:** Stability of pinosylvin in rat plasma, urine, feces, and various tissue homogenates (n=5).

Bio-sample	Nominal concentration (ng/mL)	Autosampler (24 h, 7°C)		Three freeze/thaw cycles		Room temperature (12 h)		Long term (30 days,-80°C)	
		Precision (%)	Accuracy (%)	Precision (%)	Accuracy (%)	Precision (%)	Accuracy (%)	Precision (%)	Accuracy (%)
Plasma	5.4	1.2	10.9	4.2	7.9	2.9	9.3	6	10
	135	6.4	-3.3	6.1	4.1	8.9	-3.5	9.5	-4.6
	540	8.4	0.9	9.8	0.8	8.7	4.1	6.2	1.6
Urine	1350	3.6	5	1.3	8	1.4	8.6	0.4	9.9
	5400	9.2	-1.4	5.5	-3.4	0.9	-4.7	3	-2.6
	54000	7.3	-6.8	1.6	-0.7	1.8	-6	7.3	-6.8
Feces	270	6.5	-4.4	7.8	-5.8	9.6	-3.3	12.9	8.5
	2700	2.1	-8.5	3.4	-3.2	2	7.8	2	2.7
	5400	3.8	0.7	4.7	-2.6	8.9	3.1	4.5	-1
Heart	540	1.5	9.2	4	8.3	4.7	7.7	2.6	5.4
	5400	8	-0.3	7.9	2.6	4.7	2.2	4.9	-9.6
	27000	9.6	2.1	9.1	5.7	0.5	8.7	2.3	8.2
Liver	2700	8.4	-9.6	8.4	6.8	9.6	5.2	7.2	2.8
	13500	9.7	-1.6	4	-4.9	8.3	8.7	9.2	4
	54000	6.8	-5.1	5.4	0.6	8.7	-8.1	9.4	-9.4
Spleen	270	2.4	14.7	1.6	15	1.9	17.5	1.8	16.7
	5400	7.7	2.5	4.9	4.2	6.1	-7.1	5.8	2.2
	13500	7.8	-7.5	9.2	-1.7	9.2	-7.7	6.6	-8.8
Lung	540	2.1	14.6	4.2	6	4	10.7	3.2	10.2
	2700	6.5	3.7	7.4	-5.2	4.1	11	9.5	7
	13500	9.8	2.6	6.7	4.9	6.2	5.2	9.5	8
Kidney	540	8.8	-9.1	7.2	-5.4	8.2	-6.5	9.7	1.3
	5400	9	-2.2	10.4	3	4.8	-2.7	10.8	-5.3
	13500	8	-0.3	8	-7	8.7	-1.5	9.4	1.5
Large intestine	2700	7.9	8.3	9.2	6.8	9.3	4.8	10.4	-5.4
	13500	9.5	-8.9	6.8	-8.9	3.9	-10.3	8.4	-9
	54000	8.6	5.7	8.2	8.1	7.1	8.1	7.9	7.6
Small intestine	5400	13	-2.5	8.9	-13	7.9	10.5	13.1	5.1
	108000	6.3	15.1	4.2	6.8	13.9	8.3	1.6	8.3
	216000	5.9	13.3	5	14.5	5	-4.7	4.1	-7.7
Stomach	13500	9.5	14.5	7.8	13.7	14.6	7.7	9.5	10.5
	216000	8.9	-13.7	13.3	-11.5	13.3	0.9	11.4	-0.9
	864000	13.3	-3	11.9	-9.4	11.9	-9.1	13.5	-12.2

**Table 5:** Noncompartmental pharmacokinetic parameters of pinosylvin in rats after oral administration (n=6, means ± SD).

Pharmacokinetic parameters	Unit	Value
t_1/2_	h	1.347±0.01
T_max_	h	0.137±0.016
V_d_	L/kg	434.716±25.508
CL	L/h/kg	223.635±11.866
C_max_	ng/ml	164.231±64.264
MRT_0-t_	h	3.209±0.129
MRT_0-*∞*_	h	3.210±0.129
AUC_0-24h_	ng/ml h	711.142±46.885
AUC_0-*∞*_	ng/ml h	711.195±46.885

T_1/2_: elimination half-life; T_max_: time to peak concentration; V_d_: volume of distribution; CL: clearance; C_max_: peak plasma concentration; MRT: mean retention time; AUC: area under the curve.

**Table 6:** Metabolites (M1-M9) in rats after oral administration of 49.44 mg/kg pinosylvin.

Metabolites	RT (min)	Metabolic Pathway	Mass Shift	Formula Change	Chemical Formula	[M+H]^+^	ppm
M0					C_14_H_12_O_2_	213.0865	-3.81
M1	1.20	Epoxidation	+15.99	[M+O]	C_14_H_12_O_3_	229.0859	-3.54
M2	3.03	Hydroxylation	+15.99	[M+O]	C_14_H_12_O_3_	229.0859	-2.71
M3	8.78	Methylation	+14.02	[M+CH_2_]	C_15_H_14_O_2_	227.1067	-2.49
M4	5.51	Methylation	+14.02	[M+CH_2_]	C_15_H_14_O_2_	227.1067	-1.41
M5	4.39	Double-bond Reduction	+2.02	[M+H_2_]	C_14_H_14_O_2_	215.1067	-1.98
M6	1.90	Sulfation	+79.96	[M+SO_3_]	C_14_H12O_5_S	293.0478	-1.98
M7	1.63	Acetylation	+42.01	[M+COCH_2_]	C_16_H_14_O_3_	255.1016	-2.47
M8	1.58	Glycine	+57.02	[M-OH+C_2_H_4_NO_2_]	C_16_H_15_NO_3_	270.1125	-2.63
M9	1.66	Glucuronidation	+176.03	[M+C_6_H_8_O_6_]	C_20_H_20_O_8_	389.1231	-1.06

## Data Availability

No data were used to support this study.

## References

[1] Bien-Aime S., Yu W., Uhrich K. E. (2016). Pinosylvin-based polymers: biodegradable poly(anhydride-esters) for extended release of antibacterial pinosylvin. *Macromolecular Bioscience*.

[2] Kodan A., Kuroda H., Sakai F. (2001). Simultaneous expression of stilbene synthase genes in Japanese red pine (Pinus densiflora) seedlings. *Journal of Wood Science*.

[3] Chen S., Wang L., Zhang W., Wei Y. (2015). Secondary metabolites from the root of Lindera reflexa Hemsl. *Fitoterapia*.

[4] Jiang M., Lin S., Guo Q.-L. (2013). Chemical constituents from Litsea greenmaniana. *China Journal of Chinese Materia Medica*.

[5] Fliegmann J., Schröder G., Schanz S., Britsch L., Schröder J. (1992). Molecular analysis of chalcone and dihydropinosylvin synthase from Scots pine (Pinus sylvestris), and differential regulation of these and related enzyme activities in stressed plants. *Plant Molecular Biology*.

[6] Park E.-J., Min H.-Y., Ahn Y.-H., Bae C.-M., Pyee J.-H., Lee S. K. (2004). Synthesis and inhibitory effects of pinosylvin derivatives on prostaglandin E2 production in lipopolysaccharide-induced mouse macrophage cells. *Bioorganic & Medicinal Chemistry Letters*.

[7] Jeong E., Lee H.-R., Pyee J., Park H. (2013). Pinosylvin induces cell survival, migration and anti-adhesiveness of endothelial cells via nitric oxide production. *Phytotherapy Research*.

[8] Park E.-J., Park H. J., Chung H.-J. (2012). Antimetastatic activity of pinosylvin, a natural stilbenoid, is associated with the suppression of matrix metalloproteinases. *The Journal of Nutritional Biochemistry*.

[9] Yeo S. C. M., Luo W., Wu J., Ho P. C., Lin H.-S. (2013). Quantification of pinosylvin in rat plasma by liquid chromatography-tandem mass spectrometry: application to a pre-clinical pharmacokinetic study. *Journal of Chromatography B*.

[10] Ludwiczuk A., Saha A., Kuzuhara T., Asakawa Y. (2011). Bioactivity guided isolation of anticancer constituents from leaves of Alnus sieboldiana (Betulaceae). *Phytomedicine*.

[11] Simard F., Legault J., Lavoie S., Mshvildadze V., Pichette A. (2008). Isolation and identification of cytotoxic compounds from the wood of Pinus resinosa. *Phytotherapy Research*.

[12] Park J., Pyee J., Park H. (2014). Pinosylvin at a high concentration induces AMPK-mediated autophagy for preventing necrosis in bovine aortic endothelial cells. *Canadian Journal of Physiology and Pharmacology*.

[13] Skinnider L., Stoessl A. (1986). The effect of the phytoalexins, lubimin, (-)-maackiain, pinosylvin, and the related compounds dehydroloroglossol and hordatine M on human lymphoblastoid cell lines. *Experientia*.

[14] Jiangsu New Medical College (1975). Dictionary of Chinese Medicine.

[15] Wang L.-L., Zhang Y.-B., Sun X.-Y., Chen S.-Q. (2016). Simultaneous quantitative analysis of main components in linderae reflexae radix with one single marker. *Journal of Liquid Chromatography & Related Technologies*.

[16] Sun X., Zhang Y., Chen S., Fu Y. (2016). Characterization and identification of the chemical constituents in the root of Lindera reflexa Hemsl. using ultra-high performance liquid chromatography coupled with linear trap quadrupole orbitrap mass spectrometry. *Journal of Pharmaceutical and Biomedical Analysis*.

[17] Roupe K., Halls S., Davies N. M. (2005). Determination and assay validation of pinosylvin in rat serum: application to drug metabolism and pharmacokinetics. *Journal of Pharmaceutical and Biomedical Analysis*.

[18] Roupe K. A., Yáñez J. A., Teng X. W., Davies N. M. (2006). Pharmacokinetics of selected stilbenes: rhapontigenin, piceatannol and pinosylvin in rats. *Journal of Pharmacy and Pharmacology*.

[19] Gu S., Zhu G., Wang Y. (2014). A sensitive liquid chromatography-tandem mass spectrometry method for pharmacokinetics and tissue distribution of nuciferine in rats. *Journal of Chromatography B*.

[20] Chen D.-J., Hu H.-G., Xing S.-F., Gao Y.-J., Xu S.-F., Piao X.-L. (2014). Metabolic profiling of Gynostemma pentaphyllum extract in rat serum, urine and faeces after oral administration. *Journal of Chromatography B*.

[21] Liu Y.-Q., He G.-H., Li H.-L. (2014). Plasma pharmacokinetics and tissue distribution study of roemerine in rats by liquid chromatography with tandem mass spectrometry (LC-MS/MS). *Journal of Chromatography B*.

[22] Li Y., Zeng R.-J., Chen J.-Z. (2015). Pharmacokinetics and metabolism study of isoboldine, a major bioactive component from Radix Linderae in male rats by UPLC-MS/MS. *Journal of Ethnopharmacology*.

[23] Zhu H., Liu X., Zhu T. T. (2017). UHPLC–MS/MS method for the simultaneous quantitation of five anthraquinones and gallic acid in rat plasma after oral administration of prepared rhubarb decoction and its application to a pharmacokinetic study in normal and acute blood stasis rats. *Journal of Separation Science*.

[24] Lei J. W., Guo Y. L., Bai Y., Chen S. Q. (2011). Establish the quantitative model of 3, 5-didroxystilbene in Lindera reflexa Hemsl by near-infrared spectroscopy. *Chinese Journal of Experimental Traditional Medical Formulae*.

[25] Park E.-J., Chung H.-J., Park H. J., Kim G. D., Ahn Y.-H., Lee S. K. (2013). Suppression of Src/ERK and GSK-3/*β*-catenin signaling by pinosylvin inhibits the growth of human colorectal cancer cells. *Food and Chemical Toxicology*.

[26] Li C.-Y., Qi L.-W., Li P. (2011). Correlative analysis of metabolite profiling of Danggui Buxue Tang in rat biological fluids by rapid resolution LC-TOF/MS. *Journal of Pharmaceutical and Biomedical Analysis*.

[27] Yu C., Geun Shin Y., Chow A. (2002). Human, rat, and mouse metabolism of resveratrol. *Pharmaceutical Research*.

[28] Wang H., Bai X., Sun J., Kano Y., Makino T., Yuan D. (2013). Metabolism and excretion of kakkalide and its metabolites in rat urine, bile, and feces as determined by HPLC/UV and LC/MS/MS. *Planta Medica*.

[29] Kim U., Park M. H., Kim D.-H., Yoo H. H. (2013). Metabolite profiling of ginsenoside Re in rat urine and faeces after oral administration. *Food Chemistry*.

